# Design of Saline Gel Coil for Inner Heating of Electrolyte Solution and Liquid Foods under Induced Electric Field

**DOI:** 10.3390/foods11020213

**Published:** 2022-01-13

**Authors:** Lingtao Zhang, Fan Liu, Ting Wang, Shilin Wu, Yamei Jin, Na Yang, Xueming Xu

**Affiliations:** 1 State Key Laboratory of Biobased Material and Green Papermaking, Qilu University of Technology, Shandong Academy of Science, Jinan 250353, China; 6200113234@stu.jiangnan.edu.cn (L.Z.); 7180112087@stu.jiangnan.edu.cn (S.W.); yameijin@jiangnan.edu.cn (Y.J.); 2 School of Food Science and Technology, Jiangnan University, Wuxi 214122, China; 6200113054@stu.jiangnan.edu.cn (F.L.); 6210112086@stu.jiangnan.edu.cn (T.W.); xmxu@jiangnan.edu.cn (X.X.); 3 State Key Laboratory of Food Science and Technology, Jiangnan University, Wuxi 214122, China

**Keywords:** inner heating, induced electric field, saline gel coil, aqueous electrolyte system, processing unit

## Abstract

As an emerging electrotechnology, induced electric field has attracted extensive attention in the development of innovative heat treatment equipment. In this study, a resistance heating unit based on induced electric field was built for inner heating of aqueous electrolyte solutions as well as liquid foods, such as vinegar. NaCl solutions and liquid foods with different conductivity were used to investigate the thermal effect and temperature rise of samples. Saline gel composed of 3% agar powder and 20% NaCl acted as a coil of conductor for inducing high-level output voltage. The utilization of the saline gel coil significantly improved the power conversion efficiency of the heating unit as well as the heating rate. The results revealed that duty cycle and applied frequency had immediate impact on the efficiency of inner heating. Additionally, the rate of temperature rise was proportional to the conductivity of the sample. The temperature of 200 mL NaCl solution (0.6%) increased from 25 °C to 100 °C in 3 min at 40% duty cycle and 60 kHz of applied frequency, and it was a circulating-flow process. The maximum temperature rise of black vinegar was 39.6 °C in 15 s at 60 kHz and 60% duty cycle, while that of white vinegar was 32.2 °C in 30 s under same conditions, whereas it was a continuous-flow process. This novel heating system has realized the inner heating of liquid samples.

## 1. Introduction

Liquid foods are aqueous electrolyte systems. As one of the unit operations, heat treatment is commonly applied for the inactivation of pathogenic microorganisms and detrimental enzymes in liquid foods, thus changing its physicochemical properties [1]. In these areas, the desirable effects of heat treatment are sterilization, ensuring shelf life, and eliminating harmful components, whereas the negative effects include loss of nutrients, especially heat sensitive ingredients, and an adverse impact on flavor characteristics. Conventional heat treatment is usually conducted with heat transfer, which depends on the thermal conductivity of the medium for realizing the heating [2]. Compared with inner heating, conventional methods are non-uniform heat conduction, with longer duration and lower efficiency.

Therefore, exploring novel heat treatment method has become the research focus for the food engineer. Some technologies related to electromagnetic fields have emerged, such as microwave heating [3,4], radio-frequency heating [5,6], Ohmic heating [7,8], among others. For example, Zhang et al. [9] proposed a continuous-flow microwave system for heating fluid food, and analyzed the factors affecting its efficiency. Radio-frequency treatment was also used to control pathogens of food, such as *Escherichia coli* O157:H7 and *Salmonella enterica* [10]. Both radiofrequency and microwave heating are dielectric heating processes, and their principle can be described as the process of converting electromagnetic energy into thermal energy inside the sample. The radiofrequency heating is based on the rotation of polar molecules or ions in samples, in which high-frequency electromagnetic waves promote the collision and friction of them to generate heat [11]. In addition, microwave heating is dominated by the rotation of polar molecules, and the heat is thus generated inside materials by the interactions between molecular dipoles and microwave [12]. The composition and geometry of products have a decisive effect on the heating characteristics during the process [13]. Non-uniform heat flux distribution, overheating, and unpredictable “hot” or “cold” spots resulted from electromagnetic fields have been the main challenges for industrial application of radiofrequency and microwave treatment [6,9].

Ohmic heating depends upon the conductivity of the sample and electric field strength; it is an inner heating process. According to Joule’s effect, the liquid medium to be processed acts as an electrical resistance (or the load) in the circuit; when the current passes through the interior of the sample, the electrical energy is thus converted into thermal energy [14]. However, it involves direct contact between the medium and active electrodes. Undesirable electrochemical behavior, electrode corrosion, and metal dissolution during the process have become the main issues that restrict industrial applications [15,16]. Therefore, exploring an innovative electrotechnology for inner heating of liquid foods has a potential advantage in food industry.

**Figure 1:**
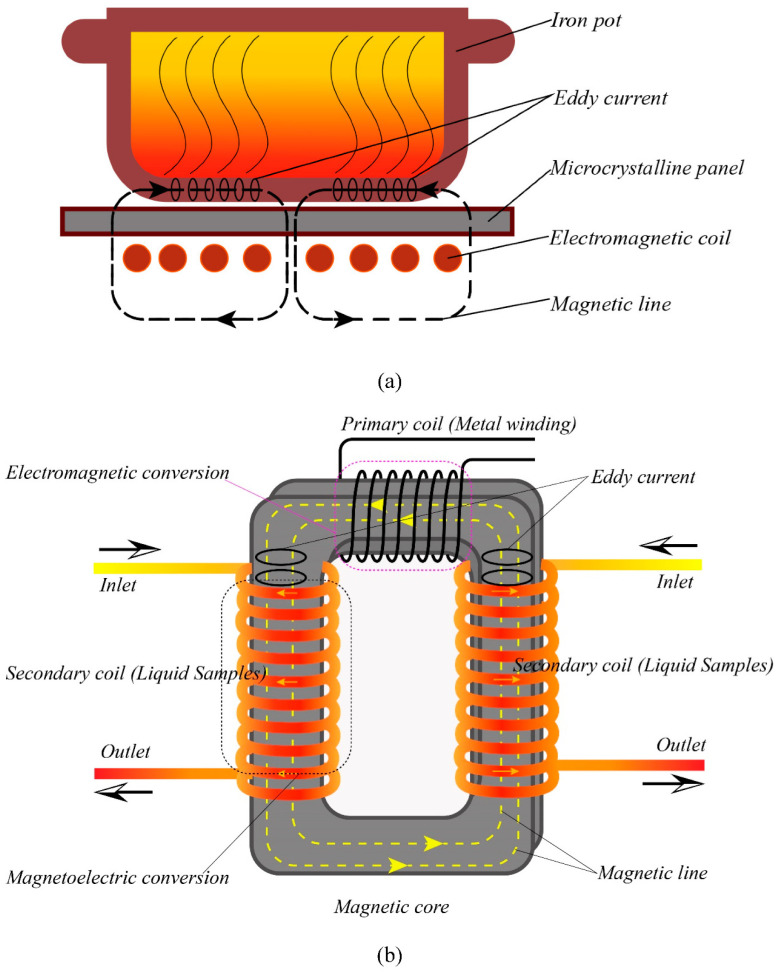
(**a**) The heating principle of induction cooker; (**b**) The heating principle of magneto-induced electric field.

Induction heating is one of the inner heating technologies, in which the alternating magnetic field is used as the source. For the treatment of foods, the process has two different ways. The first is that a magnetic field is generated when the current flows through the coil, and then eddy current and hysteresis are induced by the magnetic flux [17]. The eddy current is hindered by the extremely high resistance of vessel material; in turn, the foodstuff inside vessel is heated due to eddy current effect. The heating principle of induction cooker is based on this mechanism [18], as shown in Figure 1a. Although existing induction heating has been applied to the heating of foods, the heat transfer is still thermal conduction and convection of iron vessels [19,20]. It is not an inner heating process and thus there is no non-thermal effect on the foods. However, it is noteworthy that when the alternating magnetic field is applied on a coil of liquid foods, an induced electric field is produced in the coil. The heating process is presented in Figure 1b. Although the alternating magnetic field is also utilized, it is completely different from existing induction heating.

In a previous study, an innovative induction heating system was built [21] in which the inner heating of electrolyte solutions or liquid foods was observed. The induced voltage and temperature rise were also detected by Wu et al. [22]. However, the original system is limited by its structure and output power, thus improvement of its processing capacity, temperature rise rate, and energy utilization rate is still required. In this study, it focuses on the improvement of the system with high-frequency and high-strength electric field, as well as being equipped with a water-cooling system for magnetic circuit. The purpose is to introduce the improved system and verify its heating effect, preparing for subsequent applications. The pipeline of the coil was filled with saline gel for enhancing the electric potential difference between samples. The effects of applied frequency and duty cycle on the heating rate were also explored. Additionally, vinegar and NaCl solutions with different electrical conductivity were used as model samples to investigate the heating effect.

## 2. Materials and Methods

### 2.1. Experimental Materials

Commercial mineral water and NaCl solutions with various concentrations (0.2%, 0.4%, 0.6%, and 0.8%) were prepared. Preparation of saline gel: 200 g NaCl and 30 g agar powder were weighed, then 1 L of deionized water was added to dissolve the salt and agar powder, then the solution was heated for boiling, degassed by ultrasonic for 1 min under 40 kHz and 100 W. Immediately it was filled into the coil. In this study, agar was used as a carrier of NaCl, solidified at 37 °C into a firm gel, also known as salt bridge. During the heating, saline gel coil (salt bridge) was equivalent to a power supply, it would not be heated due to low internal resistance. Thus, agar gel would not change to liquid form. Sodium chloride and agar powder were analytical grade, purchased from Shanghai Sinopharm Chemical Reagent Co., Ltd. (Shanghai, China). White vinegar and black vinegar were supplied by Foshan Haitian flavouring & Food Co., Ltd. (Foshan, Guangdong, China).

### 2.2. Principle

**Figure 2:**
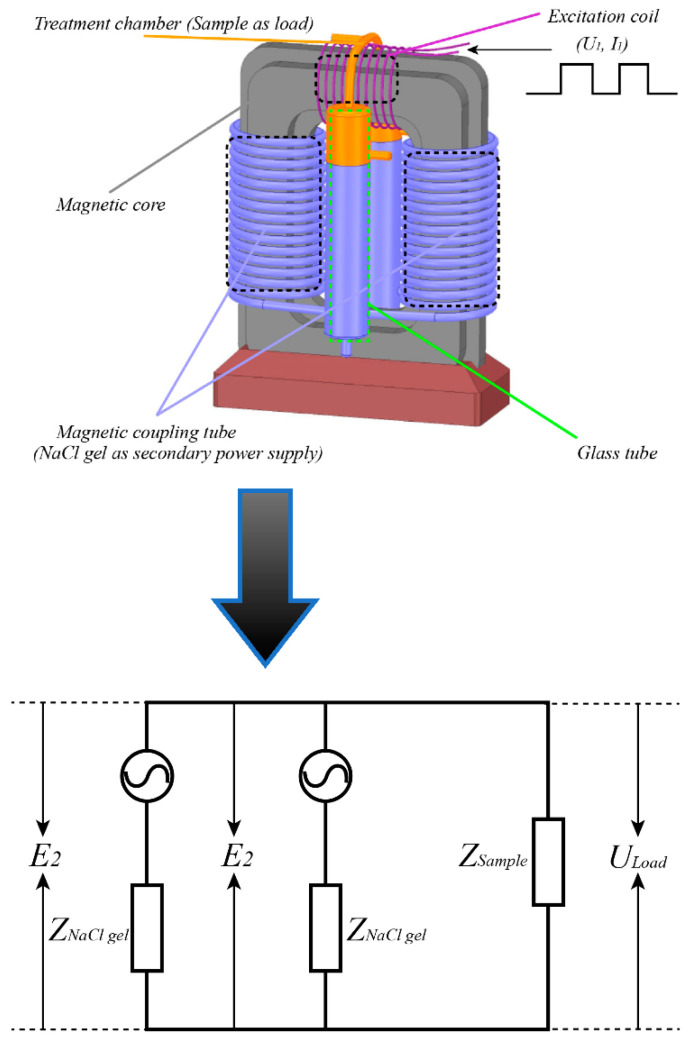
The schematic diagram of the induced electric field heating system and its equivalent circuit.

The schematic diagram of this heating system and its equivalent circuit are shown in Figure 2. According to the principle of electromagnetic induction, when the excitation voltage *U*_1_ is applied to the excitation coil, an alternating current *I*_1_ is generated, which generates alternating magnetic flux (*Φ*) in the magnetic core. Simultaneously, an induced electromotive force generated by the primary coil is *E*_1_. When the alternating magnetic flux (*Φ*) vertically passes through the magnetic coupling tube coil, the induced electromotive force *E*_2_ is generated therein. As the alternating magnetic flux (*Φ*) in the magnetic core is closed through the primary coil and the secondary coil, the induced electromotive force *E*_1_ and *E*_2_ [21] can be expressed as:

$$E_{1}=4.44fN_{1}\Phi$$

$$E_{2}=4.44fN_{2}\Phi$$

where *f* represents the applied frequency, *N*_1_ and *N*_2_ represent the turns of primary and secondary coils, respectively, and *Φ* represents alternating magnetic flux.

In theory, when conductive liquid samples are loaded on the induced electromotive force *E*_2_, an induced electric field and current would appear in the sample for the heating. The non-thermal and thermal effects originate from induced electric field strength and induced current density, respectively.

The system is based on the transformer structure. The excitation coil is wound on the magnetic core and connected to a power supply. The magnetic coupling tube is filled with saline gel to reduce the internal resistance of the system. They are located on both sides of the magnetic core, which could produce high-level electric potential difference under alternating magnetic field. In fact, they are also a power supply, then the enhanced induced voltage (or *E*_2_) is loaded on the treatment chamber for heating the samples.

### 2.3. Experiments

**Figure 3:**
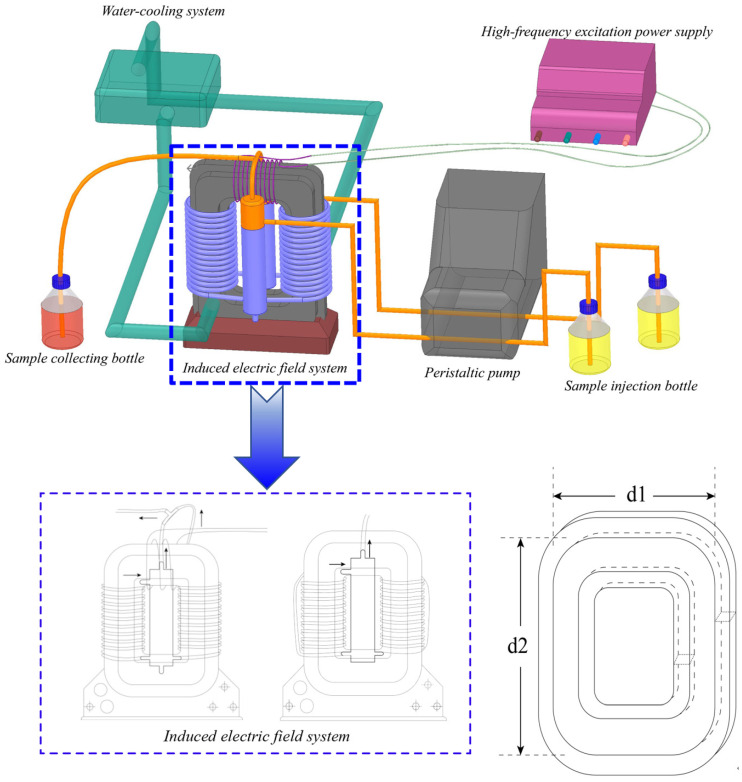
The schematic diagram and flow chart of the induced electric field heating unit (d1 = 15 cm, d2 = 25 cm).

This induced electric field heating unit includes sample bottle, collecting bottle, peristaltic pump, power supply, water-cooling system, and induction system, as shown in Figure 3. In addition, the induction system consists of a magnetic core, excitation coil, magnetic coupling pipeline, glass tube, and treatment chamber. The samples are passed through the treatment chamber for heating, then collected immediately. The parameter information, such as conductivity, applied frequency, duty cycle, among others, is presented in Table 1. It is a continuous-flow process at low flow rate as well as a circulating-flow process at high flow rate.

### 2.4. Temperature Rise and Conductivity

The measurement of temperature refers to the method of González-Monroy et al. [23] with minor modifications. At the distance of 0.3 m to the treatment chamber, the temperature was recorded via a FLIR C3 thermal camera (FLIR Systems Inc., Wilsonville, OR, USA). The conductivity was detected using a conductivity meter (FE38, Mettler Toledo Instruments Co., Ltd., Shanghai, China).

### 2.5. Statistical Analysis

All experiments were replicated in triplicate. The statistical analysis software SPSS 22.0 (IBM, Armonk, NY, USA) was used for original data analysis, and expressed as mean value ± standard deviation. All charts were drawn by Adobe Illustrator 2021 (Adobe Systems Incorporated, San Jose, CA, USA) and Origin 2019 (Origin Lab., Northampton, MA, USA).

## 3. Results and Discussion

### 3.1. Influence of Saline Gel Coil

**Figure 4:**
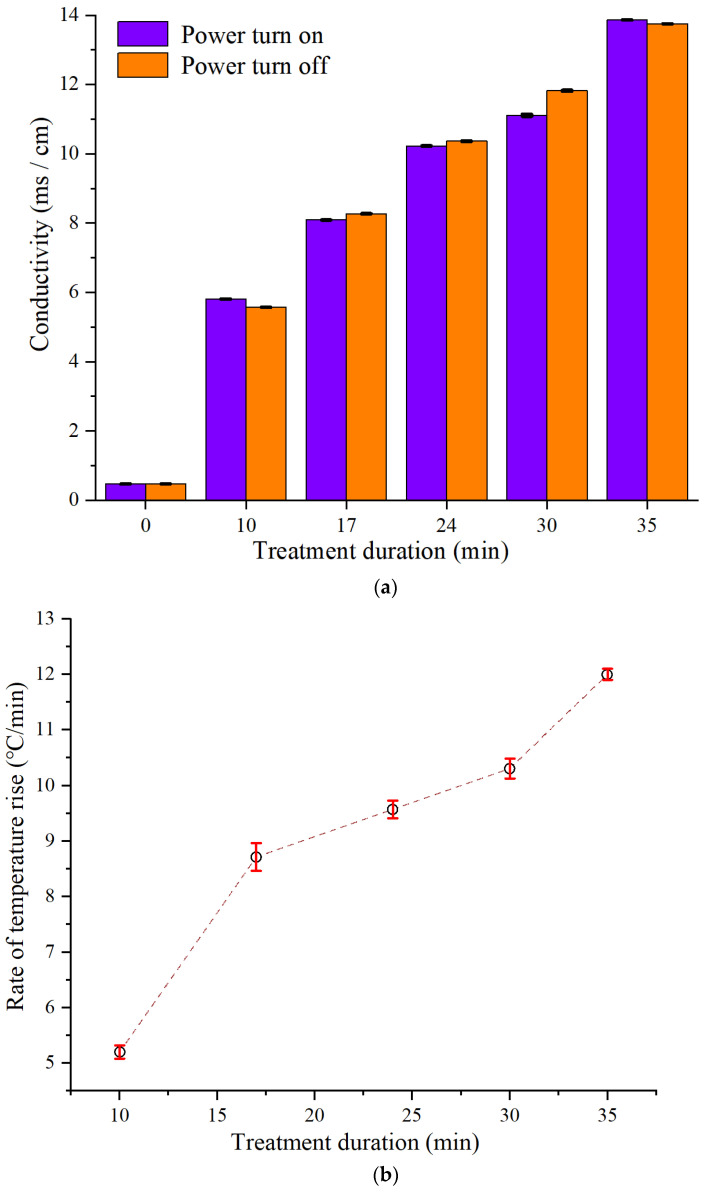
(**a**) Effect of the treatment duration on the sample conductivity; (**b**) Effect of the treatment duration on the temperature rise.

When commercial mineral water was subjected to induced electric field for 35 min, the changes of electric conductivity were shown in Figure 4a. The conductivity of the sample increased significantly after heating, which indicated that there were some Na or Cl ions in the saline coil to diffuse into the sample. Moreover, the increase in treatment duration resulted in higher conductivity, which may be ascribed to the contact duration between the sample and saline coil. Compared with the control, the conductivity of sample demonstrated the same trend, which suggested that the diffusion of Na or Cl ions was caused by the diffusion rather than alternating electric field. From the perspective of the electric field, as the periodic square wave signal with high frequency was utilized in the heating system, the induced electric field changed in both directions instead of a direct current. It implied the directional mass transfer did not occur. In another study, the electrocoalescence behaviors of water nanodroplets were observed under a direct current, confirming the direction of movement between the electrodes [24]. The temperature rise results showed that it was directly proportional to the treatment duration (Figure 4b). The reason was that the conductivity increased with the extension of duration. During the circulating-flow process, commercial mineral water with conductivity of 0.473 ms/cm rose from 25 °C to 77 °C within 10 min, the temperature rise rate was 5.2 °C/min.

**Figure 5:**
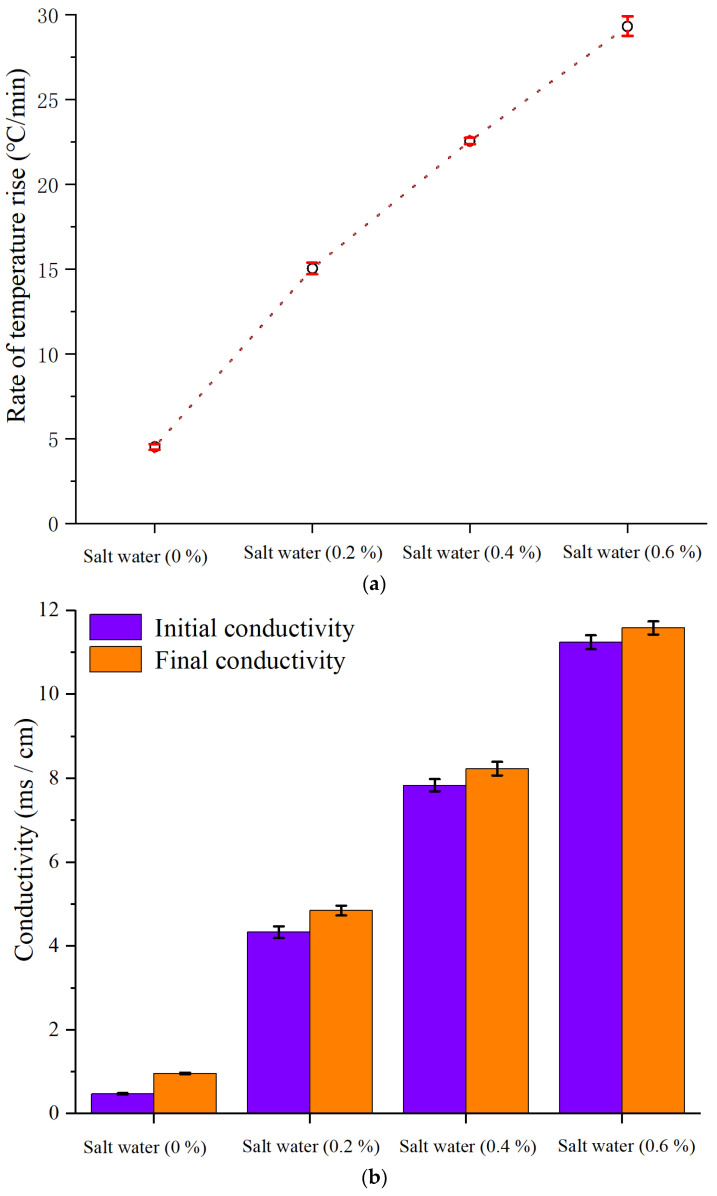
(**a**) Effect of NaCl solutions on the temperature rise; (**b**) effect of NaCl solutions on the conductivity.

The heating efficiency of the system was enhanced with the increase in conductivity (Figure 5a). In this study, 0.6% NaCl solution showed a maximum temperature rise rate of 29.3 °C/min with the cyclic treatment, and a 200 mL sample could reach boiling state within 3 min. In a previous study, the maximum terminal temperature of the sample was only 65.8 °C, and the processing capacity was limited. Compared with the initial conductivity of samples, the final conductivity increased by about 0.3–0.6 ms/cm within 10 min during the heating (Figure 5b). It was concluded the saline coil could amplify the induced electric field and improved the heating effect.

Therefore, the construction of saline coil realized the inner heating of aqueous solutions, and an extremely low level of Na or Cl ions in the saline gel coil diffused into the samples. Under an induced electric field, the main factor for ion migration was free diffusion. As the rate of the diffusion depends upon the contact duration, the diffusion of ions can be reduced by shortening the processing time. Alternatively, a buffer zone can be established to eliminate the free diffusion of ions. For various liquid food systems, the heating unit has better application prospects.

### 3.2. Influence of Physicochemical Properties

In this study, black vinegar and white vinegar with significant difference in conductivity were selected for effect validation. The conductivity of black vinegar and white vinegar were 30 ms/cm and 2.98 ms/cm, respectively. In the food conductivity category, white vinegar is equivalent to the category with lower conductivity, while black vinegar belongs to the category with higher conductivity.

**Figure 6:**
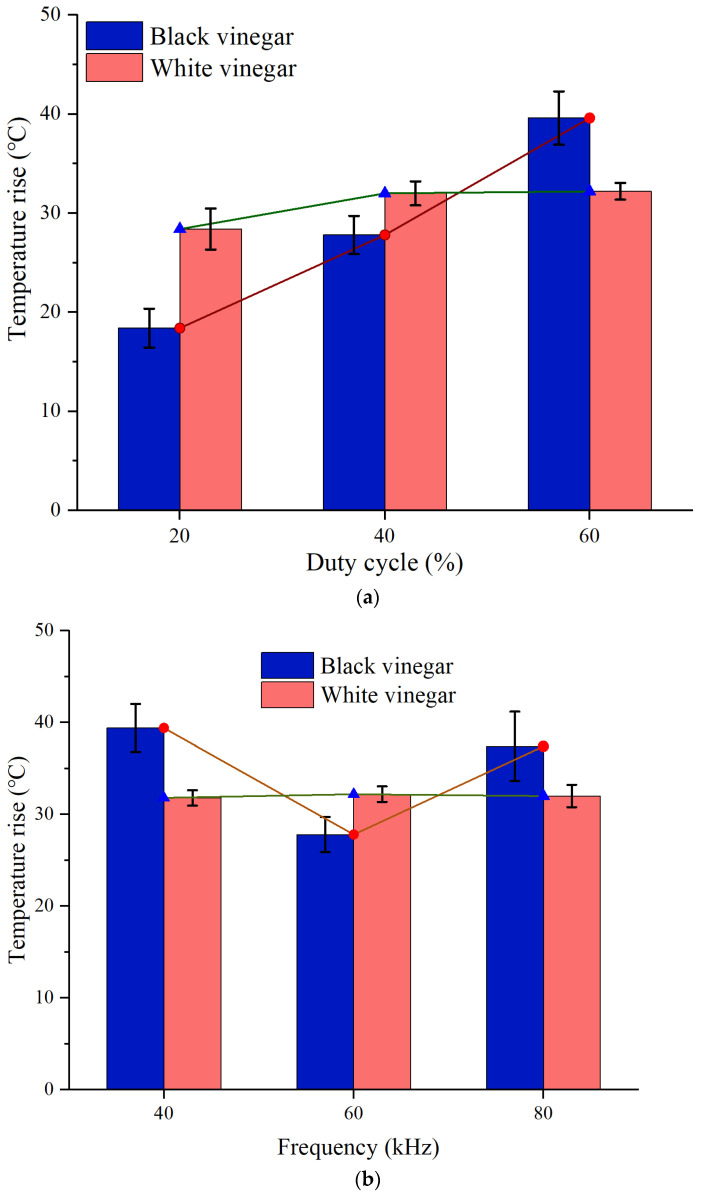
(**a**) Effect of different duty cycle on the temperature rise; (**b**) effect of different frequency on the temperature rise.

It illustrated that the temperature of black vinegar rose linearly with the increase in duty cycle (Figure 6a). Although the temperature rise trend of white vinegar was consistent with that of black vinegar, it was not obvious. There was a small discrepancy between 40% and 60% of duty cycle. For black vinegar, the increase in duty cycle resulted in a higher output power of the system, thereby the heating unit converted more electric energy into the vinegar as it passed through the treatment chamber. Due to the high conductivity of black vinegar, the losses of eddy current and hysteresis effect in magnetic core were also relatively low. For white vinegar, although the output power of the system increased, the energy conversion efficiency of the heating unit decreased due to low conductivity and large impedance of white vinegar. As a result, the eddy current effect and hysteresis effect in the magnetic core were strengthened, then the thermal energy loss increased. Additionally, the lower the conductivity of samples, the smaller the induced current in the closed circuit of the continuous-flow samples. Furthermore, the induced current density in the treatment chamber decreased, thus the temperature rise of white vinegar was relatively slow. As the current density is in the range from 0.5 to 20 A/cm^2^, the heating effect can be realized [2]. According to Ohm’s law of closed circuit, due to the existence of internal resistance of the saline coil, the sample resistance increases and the current and output voltage of the system decrease. In other words, the alternating induced electromotive force generated by the secondary power supply (or the saline coil) is limited, while the output power remains unchanged. Thereby, the electric energy is converted into thermal energy in magnetic core due to the more core loss. Contrarily, black vinegar has higher conductivity, thus the magnetic core loss is lower and the heating efficiency is relatively high.

For food systems with different electrical conductivity, the applied frequency also has a key impact on the temperature rise. As shown in Figure 6b, the temperature rise of black vinegar was significantly affected by the frequency compared to that of white vinegar, which was mainly attributed to the difference between their electrical conductivity. The temperature rise of black vinegar at 80 kHz was significantly higher than that of the sample at 60 kHz. As the impedance of secondary power supply and black vinegar diminished with the increased frequency. In turn, the induced current intensity and density were improved. This result of impedance reduction caused by higher applied frequency had been observed [25]. However, the temperature rise at 40 kHz was faster than that of 60 kHz. Lee et al. [26] reported the increased frequency in the range of 1 Hz to 20 kHz, the higher the temperature rose. The effect of frequency on the temperature rise of white vinegar was basically consistent with that of the duty cycle. Although the changes of both duty cycle and frequency influenced output power of the system, the power conversion efficiency of the heating unit had reached the consumption level due to the low conductivity of white vinegar. Therefore, the temperature rise of white vinegar is not as sensitive to the changes in parameters as black vinegar.

## 4. Conclusions

In this study, a novel magneto-induced electric field heating system has been developed. It was established according to Faraday’s law of electromagnetic induction, which has realized rapid temperature rise and uniform heat treatment of various liquid samples. Meanwhile, the saline gel coil (NaCl gel composed of 20% NaCl and 3% agar powder) was designed for reducing the internal resistance of the system and improved the energy efficiency of the heating process. Moreover, the saline gel coil did not induce NaCl diffusion into the sample under the electric field, while slight free diffusion of NaCl was observed. As the heating effect of the system was completed instantaneously, the saline gel coil structure had no adverse effect on the samples. During the heating process, the temperature rise improved with the increased sample conductivity. For effect verification, the results showed that the heating rate of sample was improved with the increased duty cycle, first decreased and then increased with the increased applied frequency. For low conductivity of the medium, the changes of duty cycle and the frequency had little effect on the heating rate. Therefore, the heating unit is consistent with Ohmic heating system. It also has the non-thermal effect due to alternating induced electric field during the treatment. This heating unit has potential application prospects for sterilization, modification, and extraction, as well as mass transfer reaction of liquid foods.

## Figures and Tables

**Table 1:** Various parameter information during processing.

Liquid Sample	Conductivity (ms/cm)	Initial Temperature (°C)	Excitation Coil Turns	Magnetic Coupling Tube Turns	Frequency (kHz)	Duty Cycle	Flow Rate (mL/min)
Commercial mineral water	0.473	25	1	30	60	40%	120
NaCl solution (0.2%)	4.333	25	1	30	60	40%	120
NaCl solution (0.4%)	7.837	25	1	30	60	40%	120
NaCl solution (0.6%)	11.24	25	1	30	60	40%	120
White vinegar	2.98	25	1	20	40, 60, 80	20%, 40%, 60%	10
Black vinegar	30.00	25	1	20	40, 60, 80	20%, 40%, 60%	20

**Note:** Flow rate 120 mL/min was a cyclic process, 10 and 20 mL/min were a one-time continuous-flow process. Duty cycle is the ratio of time a load or circuit is on compared to the time the load or circuit is off. Flow rate represents the pump speed of the peristaltic pump. Frequency represents applied frequency of power supply. Excitation coil and magnetic coupling represent the primary coil and secondary coil, respectively.

## Data Availability

The datasets used and analyzed are available from the authors.
